# Resin Transfer Moldable Fluorinated Phenylethynyl-Terminated Imide Oligomers with High T_g_: Structure–Melt Stability Relationship

**DOI:** 10.3390/polym13060903

**Published:** 2021-03-15

**Authors:** Weijie Hong, Lili Yuan, Yanping Ma, Chao Cui, Haoyang Zhang, Shiyong Yang, Wen-Hua Sun

**Affiliations:** 1 Key Laboratory of Science and Technology on High-tech Polymer Materials, Chinese Academy of Sciences, Zhongguancun, Beijing 100190, China; hongweijie@iccas.ac.cn (W.H.); zhanghaoyang@iccas.ac.cn (H.Z.); 2 School of Chemical Engineering, University of Chinese Academy of Sciences, Beijing 100049, China; 3 Key Laboratory of Engineering Plastics and Beijing National Laboratory for Molecular Sciences, Institute of Chemistry, Chinese Academy of Sciences, Beijing 100190, China; myanping@iccas.ac.cn (Y.M.); whsun@iccas.ac.cn (W.-H.S.); 4 Aerospace Research Institute of Materials & Processing Technology, Beijing 100076, China; supercc703@163.com; 5 State Key Laboratory for Oxo Synthesis and Selective Oxidation, Lanzhou Institute of Chemical Physics, Chinese Academy of Sciences, Lanzhou 730000, China

**Keywords:** polyimides, resin transfer moulding, melting stability

## Abstract

Phenylethynyl-terminated aromatic polyimides meet requirements of resin transfer molding (RTM) and exhibits high glass transition temperature (T_g_) were prepared. Moreover, the relationship between the polyimide backbones structure and their melting stability was investigated. The phenylethynyl-terminated polyimides were based on 4,4′-(hexafluorosiopropylidene)-diphthalic anhydride (6FDA) and different diamines of 3,4′-oxydianiline (3,4′-ODA), m-phenylenediamine (m-PDA) and 2,2′-bis(trifluoromethyl)benzidine (TFDB) were prepared. These oligoimides exhibit excellent melting flowability with wide processing temperature window and low minimum melt viscosities (<1 Pa·s). Two of the oligoimides display good melting stability at 280–290 °C, which meet the requirements of resin transfer molding (RTM) process. After thermally cured, all resins show high glass transition temperatures (T_g_s, 363–391 °C) and good tensile strength (51–66 MPa). The cure kinetics studied by the differential scanning calorimetry (DSC), ^13^C nuclear magnetic resonance (^13^C NMR) characterization and density functional theory (DFT) definitely confirmed that the electron-withdrawing ability of oligoimide backbone can tremendously affect the curing reactivity of terminated phenylethynyl groups. The replacement of 3,4′-ODA units by m-PDA or TFDB units increase the electron-withdrawing ability of the backbone, which increase the curing rate of terminated phenylethynyl groups at processing temperatures, hence results in the worse melting stability.

## 1. Introduction

Aromatic polyimides (PIs) are a class of engineering plastics extensively used in electronics, microelectronics and aerospace, due to their high heat resistance, excellent materials properties and electrical properties [1,2]. For the purpose of improving PIs performances and expanding their applications, various polyimide-based composites have been fabricated by doping inorganic or organic species [3,4]. Carbon fiber-reinforced polyimides composites have been widely used for high-temperature applications such as air-engines, aerospace vehicles, and precision machinery for decades [5,6,7].

Traditionally, hand lay-up prepreg/autoclave techniques have been used to fabricate polyimide/carbon fiber composites on large scales [8,9]. However, resin transfer molding (RTM) was preferred recently because of its cost advantage, better parts integration and good control of fiber volume, compared with traditional autoclave process [10]. Nevertheless, RTM process also required resins that possess a unique combination of properties. Some basic requirements could be summarized [11]: (1) before processing, the resins should be solid and fundamentally volatile-free, (2) during processing, the resins must be equipped with low and stable viscosity (<1 Pa s) to offer adequate melt flow, (3) during the processing, the material should also be of no volatile by-product and show low shrinkage.

However, due to their rigid backbones and strong interchain interaction, PIs are hard to process. It has been found that the introduction of end-capped agents at the end of polyimides backbones could limit the molecular weight of PIs and increase their processability. Polyimides terminated with many end-capped agents have been studied by many researchers, including phthalic anhydride [12], norbornene groups [13], acetylene groups [14,15], phenylethynyl groups [16,17]. Many of them can cure at high temperatures and yield crosslinked networks that can impart good heat-resistance to cured resins. Among them, phenylethynyl-terminated polyimides (PETIs) possess good processability and show good toughness and thermal stability after curing. It has been confirmed that PETIs cure by thermal induction to undergo branching, linear chain extension, cross-linking, and/or cyclization (rigidization) and/or Diels-Alder reaction without the evolution of volatile [18,19]. Phenylethynyl-terminated PETI-5 derived from 3,3′,4,4′-biphenyltetraboxylic dianhydride (s-BPDA) prepared by researchers from NASA, with a designed molecular weight of 5000 g/mol, exhibits a good combination of high fracture toughness, high heat-resistance and good processability [16]. It has minimum melting viscosity of 1000 Pa∙s at 371 °C and cured T_g_ of 270 °C. Moreover, according to the study of Scola et al., the replacement of s-BPDA units with 4,4′-(hexafluorosiopropylidene)-diphthalic anhydride (6FDA) units, resulting in a decrease in melting viscosity without much sacrifice in T_g_ [20]. The polyimide resin shows minimum melting viscosity of 48 Pa∙s at 337 °C and cured T_g_ of 268 °C. Other research also shows that polyimides synthesized by 6FDA display good thermal stability [21,22].

In recent years, significant efforts have been devoted to developing PETIs that are amenable to RTM processing. At around 2000, Connell et al. [23] have developed phenylethynyl-terminated PETI-298, PETI-330 and PETI-375 based on symmetric or unsymmetric biphenyl dianhydride (BPDA or a-BPDA), which displayed different glass transition temperatures (298, 330 and 375 °C respectively) and could be amenable to different service temperatures. All of them show low viscosity and good melting stability at 280 °C, and could be fabricated by RTM into composites with good mechanical properties [24,25]. It was also found that the replacement of s-BPDA units with a-BPDA units can equip polyimide resins with high cured T_g_ and better processability [7,26]. More recently, more unsymmetric dianhydrides have been used in the preparation of RTM resins. Polyimide resins with low melting temperature derived from 2,3,3′,4′-oxydiphthalic anhydride (a-ODPA) and 2,3,3′,4′-benzophenone anhydride(a-BTDA) terminated by phenyethynyl groups were also developed [27,28,29]. These resins could meet the requirements and could be fabricated into composites with outstanding properties. After cured at 343 °C for 8 h, the T_g_ of the formulation based on a-BTDA and 3,3′-diaminobenzophenone (3,3′-DABP) exceeded 400 °C. Furthermore, in order to improve the heat resistance of PETI, Fernberg et al. [30] introduced ethynyl moiety into the main chain of phenylethynyl end-capped polyimide resins that are amenable to RTM, by using ethynyl bis-phthalic anhydride (EBPA). During cure at 370 °C and post-cure at a temperature of over 400 °C, the T_g_ of materials could reach 400 °C. In summary, the development of polyimide resins with high T_g_ and low melting viscosity is still a great concern. The preferred polyimide resins also require a combination of properties, including good fracture toughness and no microcracks.

As mentioned above, one of the important properties of polyimide resins for RTM is their melting stability. However, to our best knowledge, there are little reports on its causes and effect factors. Usually, the viscosity of resins increases over time under isothermal conditions, which was considered to be induced by the curing of phenylethynyl groups [28,30]. Scola et al. [20]. synthesized 4-Phenylethynyl phthalic anhydride (PEPA) end-capped imide oligomers derived from 4,4′-(2,2,2-trifluoro-1-phenylethylidene) diphthalic anhydride (3FDA), 4,4′-(hexafluoroisopropylidene) diphthalic anhydride (6FDA) and 3,3′,4,4′-biphenylene dianhydride (s-BPDA), and founded 3FDA and 6FDA could endow imide oligomers better melting stability. It seems that ethynyl groups with decreased electron density show lower reactivity due to the influence of fluorine, which eventually leads to good melting stability. However, more researchers have demonstrated the cure of ethynyl of PEPA would be promoted by electron-withdrawing groups attached to phenylethynyl or ethynyl moiety. By replacing the phenyl group attached to ethynyl moiety of PEPA with naphthyl or anthracenyl group could decrease the cure temperature by 30–80 °C [31,32,33]. Harris et al. [34] reported that end groups derived from PEPA with electron-withdrawing attached to phenylethynyl moiety exhibited higher reactivity. McGrath et al. [35] designed and synthesized a series of poly(arylene ether sulfons) terminated with various phenylethynyl groups, which were attached with different substituents. It was found that the electron-withdrawing ability of the substituent group plays a major part in the cure kinetics and the reactivity of end groups increases with their electron-withdrawing abilities.

In this article, a series of low viscosity phenylethynyl end-capped oligoimides derived from 4,4′-(hexafluorosiopropylidene)-diphthalic anhydride (6FDA) with various diamine moieties were synthesized. The RTM processability, thermal properties and mechanical properties of oligoimides with different main-chain structures were compared. In this study, the electron effect of backbone on melting stability was investigated. For this purpose, the curing kinetic parameters of the oligoimides were calculated by differential scanning calorimetry (DSC), which directly influence the melting stability of oligoimides. Then, the effect of the electron effect of backbone on curing reactivity was further analyzed using ^13^C NMR and density functional theory (DFT) method.

## 2. Materials and Methods

### 2.1. Materials

2,2′-bis(trifluoromethyl)benzidine (TFDB) were purchased from Chinetech (Tianjin, China) Chemical Co. Ltd and used as received. 4,4′-(hexafluorosiopropylidene)-diphthalic anhydride (6FDA) (Chinetech Chemical Co, Tianjin, China) was dried in a vacuum oven (Shanghai Yiheng Scientific Instrumrent Co, Shanghai, China) at 150 °C for 12 h prior to use. 3,4′-oxydianiline(3,4′-ODA) and m-phenylenediamine (m-PDA) were purchased from. Changzhou Sunlight Pharmaceutical Co. Ltd. (Changzhou, China) and used as received. 4-phenylethynyl phthalic anhydride (PEPA) (Changzhou Sunlight Co, Changzhou, China) was dried in a vacuum oven at 110 °C for 12 h before use. N-methyl pyrrolidone (NMP) was supplied by Beijing Beihua Fine Chemicals (Beijing, China) and purified by distillation over P_2_O_5_. Toluene, isoquinoline and ethanol were purchased from China National Medicines Corp. (Beijing, China) and used as received.

### 2.2. Synthesis of Model Compounds

**Scheme 1:**
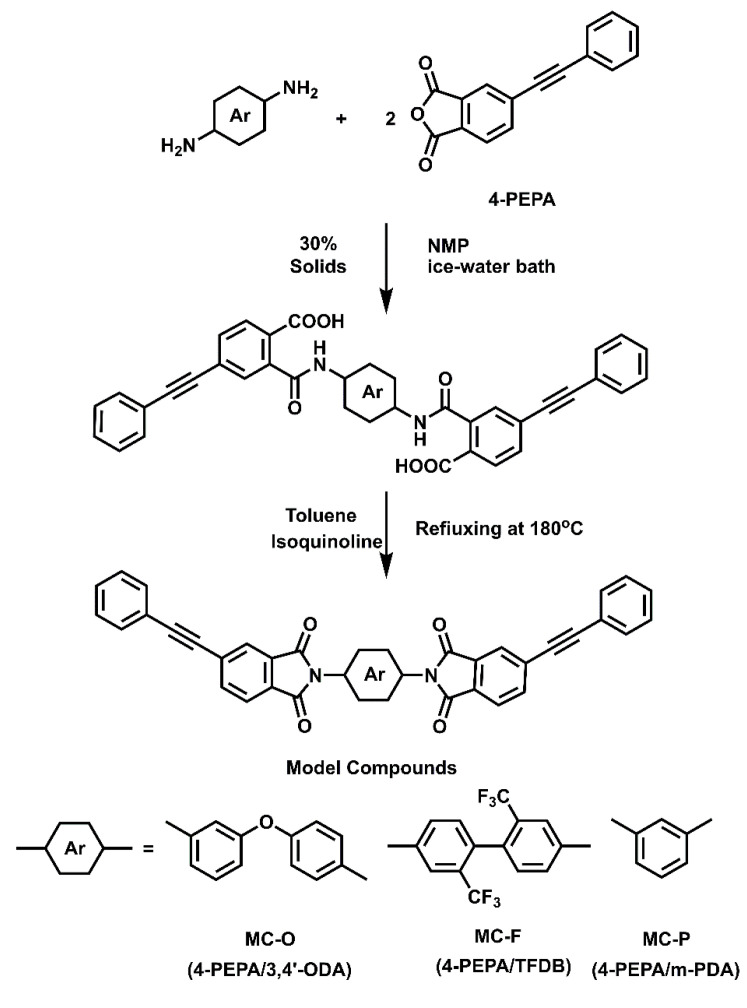
Synthesis of the model compounds.

Model compounds derived from 3,4′-ODA (MC-O), m-PDA (MC-P) and TFDB (MC-F) were prepared by one-pot high-temperature polymerization (Scheme 1). In a typical experiment, MC-O was prepared in the following procedure: 3,4′-ODA (12.34 g, 60.54 mmol), PEPA (30.06 g, 121.1 mmol) and 98.42 g NMP were placed into a 250 mL flask equipped with a condenser, a mechanical stirrer (IKA, Guangzhou, China), a thermometer (Zhongxing Glass Gage, Wuqiang, Hebei, China), a dean stark trap and nitrogen flow. The reaction solution was stirred for 18 h in an ice-water bath. 9.89 g toluene and a catalytic amount of isoquinoline were added. The mixture was heated to 180 °C for 10 h. The flask was removed from the heat, cooled in an ice-water bath, and the dark-brown solution was poured into a high-speed laboratory blender (IKA, Guangzhou, China) containing adequate boiling water/ethanol (*v*/*v* = 1:1) mixture to precipitate MC-O crude product. The fine powder was isolated by filtration and washed with boiling water/ethanol twice. The solid powder was dried at 200 °C under vacuum for 12 h to obtain a yellow powder (38.12 g, yield: 95.3%). m.p, (DSC): 290 °C. ^1^H NMR (CF_3_COOD, δ ppm): 8.13 (d, 2H), 8.01 (d, 4H), 7.55–7.61 (m, 5H), 7.36–7.39 (m, 8H), 7.20–7.29 (m, 5H). FTIR (KBr cm^−1^): 2210 (−C≡C−), 1777 (asym C=O str), 1722 (sym C=O str), 1372 (imide C−N str), 1244 (asym C−O−C str), 1096 (sym C−O−C str). Elem. Anal. Calcd for C_44_H_24_N_2_O_5_: C, 79.99%; H, 3.66%; N, 4.24%. Found: C, 79.90%; H, 3.73%; N, 4.20%.

The model compound MC-P was prepared according to the above method except that 3,4′-ODA was replaced by m-PDA to obtain yellow powder (38.80 g, yield: 97.0%). m.p, (DSC): 279 °C. ^1^H NMR (CF_3_COOD, δ ppm): 8.05 (s, 2H), 7.93 (s, 4H), 7.66–7.71 (m, 1H), 7.61 (s, 1H), 7.54–7.57 (d, 2H), 7.46–7.48 (m, 4H), 7.27–7.29 (d, 6H). FTIR (KBr cm^−1^): 2211 (−C≡C−), 1777 (asym C=O str), 1732 (sym C=O str), 1362 (imide C−N str). Elem. Anal. Calcd for C_38_H_20_N_2_O_4_: C, 80.27%; H, 3.55%; N, 4.93%. Found: C, 80.40%; H, 3.59%; N, 4.96%.

The model compound MC-F was prepared according to the method as described above except that 3,4′-ODA was replaced by TFDB to obtain red brown powder (38.51 g, yield: 96.3%). m.p, (DSC): 290 °C. ^1^H NMR (CF_3_COOD, δ ppm): 8.19 (s, 2H), 8.07 (s, 4H), 7.96 (s, 2H), 7.77 (d, 2H), 7.60–7.96 (m, 6H), 7.41 (s, 6H). FTIR (KBr cm^−1^): 2213 (−C≡C−), 1778 (asym C=O str), 1729 (sym C=O str), 1364 (imide C−N str), 1073–1177 (C−F str). Elem. Anal. Calc’d for C_46_H_22_F_6_N_2_O_4_: C, 70.77%; H, 2.84%; N, 3.59%. Found: C, 71.18%; H, 3.02%; N, 3.70%.

### 2.3. Preparation of Oligoimides End-Capped with Phenylethynyl

**Scheme 2:**
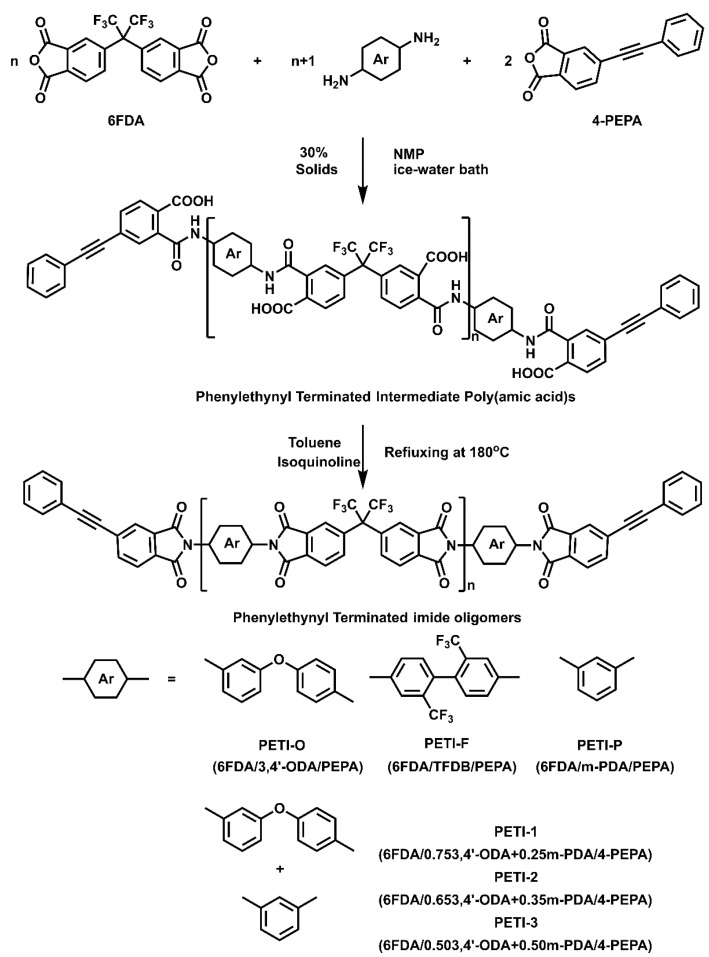
Synthesis of fluorinated phenylethynyl-endcapped polyimides.

The oligoimides with designed M_n_Cs of 1000 g/mol were also prepared by the one-pot high-temperature polymerization with the reaction of 6FDA, 4-PEPA and different aromatic diamines (Scheme 2). A typical preparation procedure of PETI-O is illustrated below. To a 250 mL three-neck round bottom flask with a condenser, mechanical stirrer, thermometer, dean stark trap and nitrogen flow was added 6FDA (15.46 g, 34.8 mmol) and NMP (66.0 g). The mixture was stirred at room temperature until the aromatic dianhydrides were completely dissolved. Subsequently, 3,4′-ODA (17.9 g, 89.2 mmol) and about 10 g NMP was added. The mixture was then stirred for 6 h in an ice-water bath. 4-PEPA (27.0 g, 10.9 mmol) and residual NMP were added into the flask, resulting in a reaction mixture with 30% solids (*w*/*w*). The mixture was stirred in an ice bath for another 16 h. 13.56 g toluene and a catalytic amount of isoquinoline were added. The mixture was heated to 180 °C for 10 h. For the purpose of elimination of toluene, the mixture was heated to 200 °C. The flask was then cooled by ice-water bath, and the dark brown solution was poured into a high-speed laboratory blender containing adequate boiling water/ethanol (*v*/*v* = 1:1). The precipitates were isolated by filtration and washed with boiling water-ethanol twice. The solid was dried at 200 °C under vacuum for 12 h to obtain a yellow powder (53.1 g, yield: 97%).

### 2.4. Preparation of the Thermally-Cured Polyimides

The oligoimide powders were compression moulded in stainless steel (80 × 80 mm) with a hot-press machine (IDM Co, Washington DC, USA). The oligoimide powders were melted at 305 °C. The temperature was gradually raised to 380 °C in 50 min. The pressure of 4 MPa was applied, the powders were cured for 2 h. When the cured resins were cooled to about 250 °C, the pressure was released. The PI sheets (80 × 80 × 2 mm) were removed from the mould and stored under ambient temperature.

### 2.5. Measurements

Infrared spectra (IR) were measured on a Perkin-Elmer 782 Fourier transform infrared (FTIR) spectrometer (Perkin-Elmer, Waltham, MA, USA). ^1^H and ^13^C NMR spectra were obtained on a Bruker AVANCE 400 spectrometer (Bruker, Karlsruhe, Germany) using dimethyl sulfoxide (DMSO-d_6_) or trifluoroacetic acid (CF_3_COOD) as a solvent. Gel permeation chromatography (GPC) was performed on a Waters system equipped (Waters, Milford, MA, USA) with a model 1515 pump, a 2414 refractive index detector using NMP containing LiBr (J$K Co, Beijing, China, 0.02 mol/L) as the eluant. The elemental analysis was carried out on a Thermo Flash Smart elemental analyzer (Thermo Fisher Scientific, Carlsbad, CA, USA).

Matrix-assisted laser desorption/ionization time of flight (MALDI-TOF) mass spectra were carried out with an Autoflex IIIMALDI-TOF mass spectrometer (Bruker, Karlsruhe Germany). Wide-angle X-ray diffraction (WXRD) measurements were taken on a Rigaku D/max-2500 X-ray powder diffractometer (Riguku Beijing Co, Beijing, China) with Cu/Kα radiation, operated at 40 kV and 200 mA. The 2θ scan data were collected in the scale of 3°–60°. The geometry optimization, Natural bond orbital charge distribution and spin density distribution were achieved by Gaussian09, Revision E.01 program (Gaussian Inc, Wallingford, CT, USA) using the M062X/6-311+G** basis sets.

Differential scanning calorimetry (DSC) analysis was performed on a TA-Q100 analyzer (TA Instruments, New Castle, DE, USA) at a heating rate of 10 °C/min under nitrogen atmosphere. Thermogravimetric analysis (TGA) was accomplished on a TA-Q50 series thermal analysis system (TA Instruments, New Castle, DE, USA) at a heating rate of 20 °C/min under nitrogen or air atmosphere. Complex viscosity (|η*|) was performed on a TA-AR2000 rheometer equipped (TA Instruments, New Castle, DE, USA) with a 25 mm-diameter parallel plate fixture measured at the heating rate of 4 °C/min. The upper plate was oscillated at a fixed strain of 0.1% and a constant angular frequency of 5 rad/s, while the lower one was attached to a transducer that recorded the resultant torque and then converted to the complex viscosity. Dynamic mechanical analyses (DMA) were performed on a TA-Q800 analyzer (TA Instruments, New Castle, DE, USA) with a heating rate of 5 °C/min. A three-point bending mode was employed with the specimen size of 20 × 5 × 2 mm.

The mechanical characteristics of the thermally-cured resins were determined at 25 °C via an Instron 5567 universal testing machine (Instron, Norwood, MA, USA), and the resultant values represented the average of at least five runs per specimen. Tensile strength and modulus of the 5.0 mm-wide sample were acquired at a crosshead speed of 1.0 mm/min following GB/T 1040-2006, and flexural strength and modulus of 3.0 mm-wide one were obtained at a rate of 1.0 mm/min following HG/T 3840-2006, respectively.

### 2.6. Cure Kinetics of Phenylethynyl-Endcapped Oligoimides

The analyses of oligoimides and the measurement of T_g_ at different thermally-heated times and temperatures were accomplished by the DSC method (TA-Q100) under a nitrogen atmosphere at a flow rate of 40 mL/min. Temperatures were calibrated by indium and zinc standards.

In order to calculate the activation energy E_a_ of the phenylethynyl curing reaction, Kissinger’s method [36,37,38,39] was used. The uncured imide oligomers were scanned by DSC at different heating rates (2, 5, 10, 15 and 20 °C/min, respectively). The exothermic peak temperatures (*T*_p_) increase with the ramping of the heating rate (β). Usually, it was assumed that the reaction rate reaches the maximum at peak temperatures. Hence, Equation (1) can be obtained.

(1) $$\ln\frac{\beta }{T_{p}^{2}}=\ln\frac{\mathrm{AR}}{E_{a}}+\ln\left[{-f}^{’}\left(\alpha_{p}\right)\right]-\frac{E_{a}}{{\mathrm{RT}}_{p}}$$

where A is the pre-exponential factor; E_a_ is the activation energy, R is the gas constant, f’(α_p_) is the differentiation of the function of convention. The value of E_k_ can be calculated from the slope of the plot of $\ln\left(\beta /T_{p}^{2}\right)$ vs. 1/T_p_.

Samples with a mass of about 7 mg in size were heat-treated at 290 °C or 300 °C for different times. The effects on T_g_ by altering of temperature or time were investigated. In order to eliminate the effect of possible crystalline on the T_g_ values, samples were quenched before the measure of T_g_ values. The scan of the samples was firstly carried out from 40 to 300 °C at a rate of 20 °C/min; after being cooled quickly to 40 °C, the samples were heated to 500 °C at the rate of 10 °C/min. The modified DiBenedetto equation was used to calculate the reaction extent (x) by determining the T_g_ for a highly crosslinked network [36,40,41,42].

(2) $$\frac{T_{g}{-T}_{g0}}{T_{g\infty }{-T}_{g0}}=\frac{\lambda x}{1-(1-\lambda )x}$$

where T_go_ and T_g__∞_ represent the glass transition temperatures before cure and after full cure. T_g_ means the glass transition temperature of the sample isothermally cured at each temperature for specific times. λ is equal to the ratio of the complete cured material’s isobaric heat capacity of, ΔC_p∞_, to that of uncured material, ΔC_p0_. Experimentally, λ could be calculated as follows [42].

(3) $$\lambda =\frac{\Delta C_{p\infty }}{\Delta C_{p0}}=\frac{T_{g0}}{T_{g\infty }}$$

In addition, even if the selected T_g∞_ does not correspond to the ideal value of fully cured materials, the calculated x is still valid for kinetic analysis. In this case, x is substituted by x’ that refers to x/x_M_, a relative reaction extent. In this study, T_g_s of resins cured at 380 °C for 2 h were defined as T_g∞_s.

## 3. Results and Discussions

### 3.1. Characterization of Oligoimides End-Capped with Phenylethynyl Groups

A series of phenylethynyl end-capped aromatic oligomers derived from 6FDA was prepared via one-pot high-temperature polymerization (Scheme 2). PETI-O, PETI-F and PETI-P were prepared by reacting 6FDA with three different diamines. PETI-1, PETI-2 and PETI-3 were prepared by reacting 6FDA with a mixture of 3,4′-ODA and m-PDA at different mole ratios. The molecular weights of oligoimides were measured by GPC. As showed in Table 1, all oligoimides showed similar M_n_ of about 1300 g/mol, which were close to the calculated molecular weight of 1000 g/mol.

**Figure 1:**
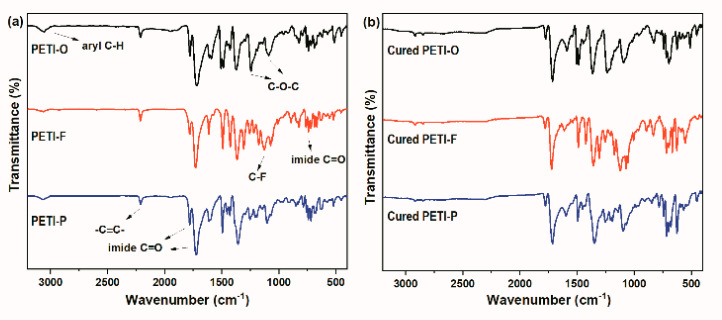
Representative FT-IR spectra of (**a**) phenylethynyl-terminated polyimides (PETIs) and (**b**) the corresponding cured PETIs.

Figure 1 shows the representative FTIR spectra of PETI-O, PETI-F and PETI-P before and after thermally cured at 380 °C for 2 h. The characteristic absorption at 2212 cm^−1^ attributed to stretching vibrations of ethynyl (–C≡C–) groups and the absorption at 3059 cm^−1^ attributed to aromatic C–H stretching vibration are observed. The asymmetric and symmetric absorption peaks at 1780 and 1722 cm^–1^, as well as the bending vibration peak at 743 cm^–1^ are attributed to the imide C=O in the oligomer backbone. Meanwhile, the absorption at 1360 cm^–1^ is assigned to the vibration of C–N stretching. Moreover, the asymmetric and symmetric absorption peaks at 1244 and 1103 cm^–1^ assigned to C–O–C stretching vibration for PETI-O, and 1124 cm^–1^ assigned to C–F stretching vibration for PETI-F are also detected. The cured oligoimides show no absorption at 2212 cm^–1^, demonstrating that phenylethynyl groups were consumed during the thermal-curing process.

The solution ^1^H NMR and ^13^C NMR spectra of representative PETI-O, PETI-F and PETI-P are presented in Figures S1–S3. The peaks are shown in ^1^H NMR and ^13^C NMR assigned as expected, which clearly recognize the designed chemical structures of samples, indicating the objective compound. All signals of carbonyl carbons are observed at 166 ppm in ^13^C NMR. The signal detected in about 7.50 ppm is attributed to the C_1,2_ in ^1^H NMR. The resonances of the ethynyl carbon atoms are observed at 86 ppm and 95 ppm in ^13^C NMR. 

**Figure 2:**
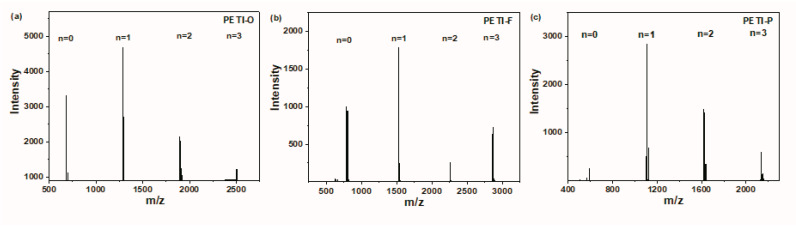
(**a**) MALDI-TOF mass spectrum of PETI-O; (**b**) MALDI-TOF mass spectrum of PETI-F; (**c**) MALDI-TOF mass spectrum of PETI-P.

Molecular structures of the representative PEPA end-capped oligoimides of PETI-O, PETI-F and PETI-P were also characterized by MALDI-TOF. Figure 2 shows the result, and the resin species detected are listed in Table S1. All the PETIs mainly consist of 4 different components with different polymerization degrees (*n* = 0, 1, 2, 3). The molecular weights of the resin species detected were in good accordance with the calculated ones. For instance, PETI-O has a peak located at *m*/*z* = 1291 corresponding to the species with *n* = 1, which shows the highest peak intensity. The main species located at 1532 *m*/*z* and 1108 *m*/*z* with *n* = 1 were also detected in the samples of PETI-F and PETI-P, respectively. Clearly, the characterization of MALDI-TOF demonstrated the accuracy of syntheses.

### 3.2. Melt Fluidity of Phenylethynyl-Endcapped Oligoimides

**Figure 3:**
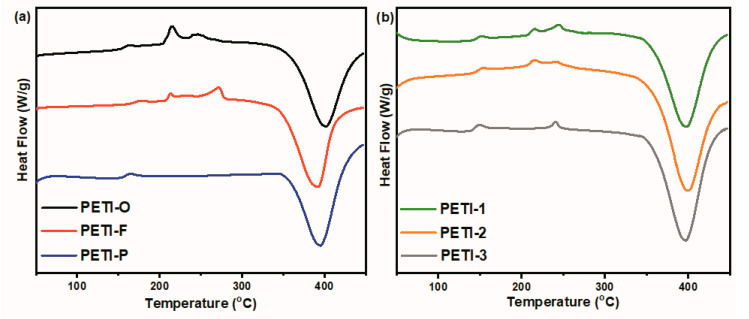
DSC curves of (**a**) PETI-O, PETI-F, PETI-P and (**b**) PETI-1, PETI-2, PETI-3.

Figure 3 compares the DSC curves of different oligoimides, and Table 2 summarizes the DSC data of them. It is noteworthy that PETI-F exhibited the highest T_g_ of 170 °C, which is caused by rigidity and linearity biphenyl moiety substituted by –CF_3_ groups [43,44], while PETI-O and PETI-P show similar T_g_ values. Meanwhile, apparent melting endotherms are observed for PETI-O and PETI-F, while PETI-P shows no melting behavior. The exothermal peaks in the range of 350–450 °C are due to the thermal curing reaction of phenylethynyl. PETI-F shows the lowest curing onset temperature of 350 °C and peak-temperature of 391 °C. PETI-O and PETI-P have similar curing onset temperatures; however, PETI-P shows a lower T_exo_ of 395 °C. It indicates that the curing reactivity of the three PETIs increases in the order: PETI-O < PETI-P < PETI-F. It seems that the m-PDA and TFDB moieties in polymer backbones encourage the curing reaction compared with 3,4′-ODA moiety.

PETI-1, PETI-2 and PETI-3 show T_g_ of 144–148 °C. It was also found that the introduction of m-PDA decreases the crystalline of oligoimides. The melting enthalpy of oligoimides decreased from 38.7 J/g of PETI-O with no m-PDA moieties in its main chains to 6.30 J/g of PETI-3, which was synthesized by 3,4′-ODA and m-PDA at a mole ratio of 50:50. Obviously, the introduction of m-PDA units decreases the crystallinity of PETIs. PETI-1, PETI-2 and PETI-3 have curing onset temperatures of 357–361 °C and peak temperatures of 397–401 °C.

**Figure 4:**
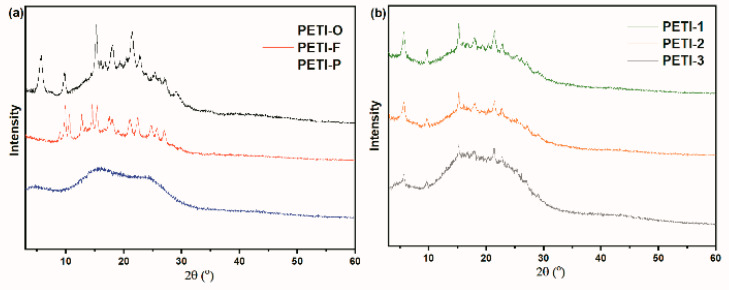
Wide angle X-ray diffraction patterns of (**a**) PETI-O, PETI-F, PETI-P and (**b**) PETI-1, PETI-2, PETI-3.

The WXRD patterns of oligomers are shown in Figure 4. Because of the difference in chemical structure, PETI-F shows unique crystalline characteristics. PETI-O shows obvious diffraction peaks, while the WXRD pattern of PETI-P is broad, revealing its amorphous nature. It has been demonstrated that unsymmetrical structures and meta catenation in polyimides backbones would increase their solubility and melt processability. The bent chains endowed by m-PDA decrease the packing of oligomer chains, hence tend to decrease interactions between the oligoimide chains, which lead to the decrease of the crystallinity of oligoimides [45,46]. Other oligoimides copolymerized by 3,4’-ODA and m-PDA displayed semi-crystalline behavior to different extent with diffraction peaks between 5°–30°. It is distinct that as the increase of m-PDA moieties content in the main chains, the diffraction peaks gradually become smooth. It implies the crystallinity of PETIs decrease due to the introduction of m-PDA, which is in consistence with the above DSC analysis.

**Figure 5:**
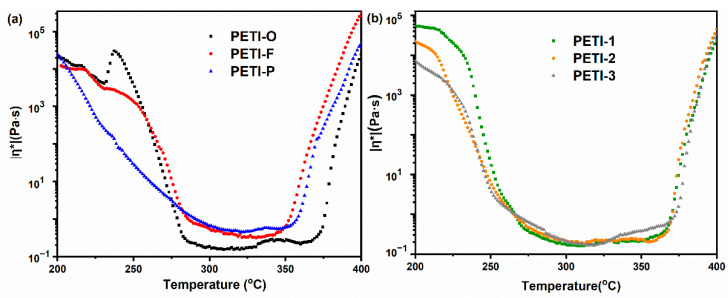
Complex melt viscosities of the (**a**) PETI-O, PETI-F, PETI-P and (**b**) PETI-1, PETI-2, PETI-3

In order to investigate the melting flowing ability of the PEPA end-capped oligoimides, rheology behaviors of them were characterized, whose detailed results are displayed in Figure 5 and Table 2. Due to the presence of hexafluoroisopropylidene (–C(CF_3_)_2_–) units with bulky trifluoromethyl in the backbone, which reduce chain packing or lower the stiffness of polymer chain, all oligoimides are provided with low values of minimum viscosities (min |η*| < 1 Pa·s) [47,48]. All the rheological curves show similar patterns. For instance, with ramping of temperature, the complex viscosity of PETI-O firstly descends significantly. After temperature values grow higher than 280 °C, the viscosity values keep low (< 1 Pa·s) until the temperature values reach 375 °C. Then the viscosity value ascends significantly due to the crosslinking of phenylethynyl groups. Herein, temperature scale with |η*| < 1 Pa·s (ΔT_|η*|__≤1 Pa∙s_) is defined as processing temperature window for RTM. PETI-O, PETI-F and PETI-P all have low ΔT_|η*|__≤1 Pa∙s_ of 95 °C, 65 °C and 65 °C, respectively. With the rigid diamine moieties of TFDB and m-PDA contained by PETI-F and PETI-P, they show worse chain movement ability and higher minimum viscosities of 0.31 Pa∙s at 331 °C and 0.45 Pa∙s at 323 °C. Compared with PETI-P, the lower minimum melt viscosity for PETI-F may be caused by the non-coplanar twist-biphenyl structure of TFDB and increased free volume imparted by –CF_3_ groups, which disrupt the chain packing and decrease the interactions between the oligoimide chains for PETI-F [43,49,50]. PETI-1, PETI-2 and PETI-3 copolymerized by 3,4′-ODA and m-PDA show lower crystallinity than PETI-O and PETI-F, having lower melting temperature. Therefore, They also gain wider processing temperature windows with ΔT_|η*|__≤1 Pa∙s_ of 102 °C, 104 °C and 105 °C, respectively. Although PETI-1, PETI-2 and PETI-3 are different from the content of rigid m-PDA moieties in main chains, they have similar minimum viscosities (0.16 Pa∙s ~ 0.18 Pa∙s), which are still higher than that of PETI-O (0.15 Pa∙s at 309 °C).

### 3.3. Melting Stability of the Phenylethynyl Endcapped Oligoimides

**Figure 6:**
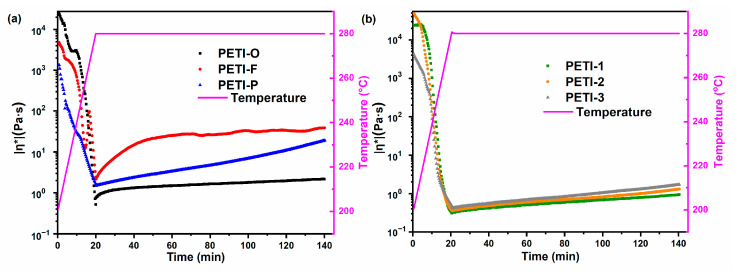
The variation of complex viscosities of (**a**) PETI-O, PETI-F, PETI-P and (**b**) PETI-1, PETI-2, PETI-3 at 280 °C.

**Figure 7:**
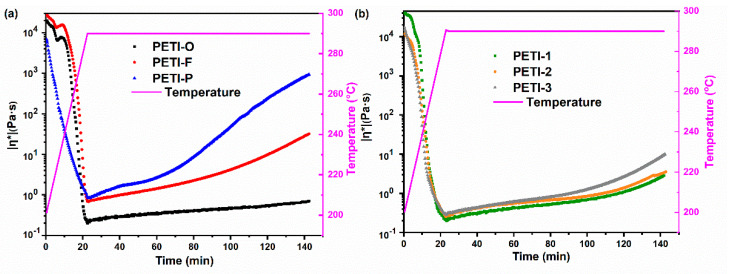
The variation of complex viscosities of (**a**) PETI-O, PETI-F, PETI-P and (**b**) PETI-1, PETI-2, PETI-3 at 290 °C.

**Figure 8:**
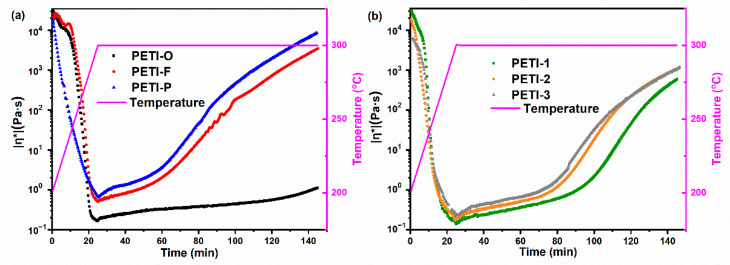
The variation of complex viscosities of (**a**) PETI-O, PETI-F, PETI-P and (**b**) PETI-1, PETI-2, PETI-3 at 300 °C.

The complex viscosity(|η*|) of the oligoimides as a function of time at 280 °C (Figure 6), 290 °C (Figure 7) and 300 °C (Figure 8) are displayed in Figure 6, Figure 7 and Figure 8, respectively; and the detailed data is shown in Table 2.

For oligimides with different chemical structures of main chains, their variations of |η*| during the isothermal heating are also different. For instance, PETI-P has the worst melting stability with viscosity variation of 0.88–930 Pa∙s at 290 °C, compared with PETI-O (0.20–0.70 Pa∙s) and PETI-F (0.69–32.0 Pa∙s). The variation of melting viscosity of the three PETIs increased in the order of PETI-O < PETI-F < PETI-P.

By copolymerization of 3,4’-ODA and m-PDA, PETI-1, PETI-2 and PETI-3 gain medium melting stability in comparison with PETI-O and PETI-P. The more content of m-PDA moieties, the worse melting stability of oligoimides. PETI-1 has the lowest viscosity variation of 0.31–0.94 Pa∙s at 280 °C, compared with PETI-2 (0.35–1.31 Pa∙s) and PETI-3 (0.46–1.72 Pa∙s), exhibiting the best processability. In summary, PETI-O and PETI-1 can meet requirements of RTM at 290 and 280 °C, respectively.

As mentioned in the introduction section, the variation of viscosity of oligoimides was considered to relate to the cure of phenylethynyl groups. As mentioned in Introduction section, phenylethynyl groups undergo complex linear chain extension, branching, cross-linking and/or cyclization (rigidization) during the heating process, which leads to the increase of molecular weight. The polymer melt viscosity extremely depends on the molecular weight. There is a well-known empirical power law between the zero-shear rate viscosity η_0_ and weight average molecular weight M_w_ [51,52,53].

η_0_ = KM_w_^α^ (4)

K is a constant, which is related to the property of the polymer. When M_w_ is lower than a critical value (M_c_), polymer chain cannot form effective entanglement, whose α is 1.0. If the polymer has higher M_w_, α is 3.4. It is reasonable to believe that the variation of viscosity of PETIs is related to their curing rates, melting flow properties and entanglement properties. The different melting stability of the synthesized oligoimides is resulted from their different chemical structures, which will be discussed in the next section in combination with more evidence of kinetic experiment.

### 3.4. The Effect of Chemical Structures on Melting Stability

#### 3.4.1. Investigation of Cure Kinetics of Phenylethynyl Endcapped Oligoimides

In order to explore the effect of chemical structures on melting stability, the curing reactivity of oligoimide with different chemical structures was compared firstly. PETI-O, PETI-F and PETI-P were chosen to carry out the kinetic analysis.

**Figure 9:**
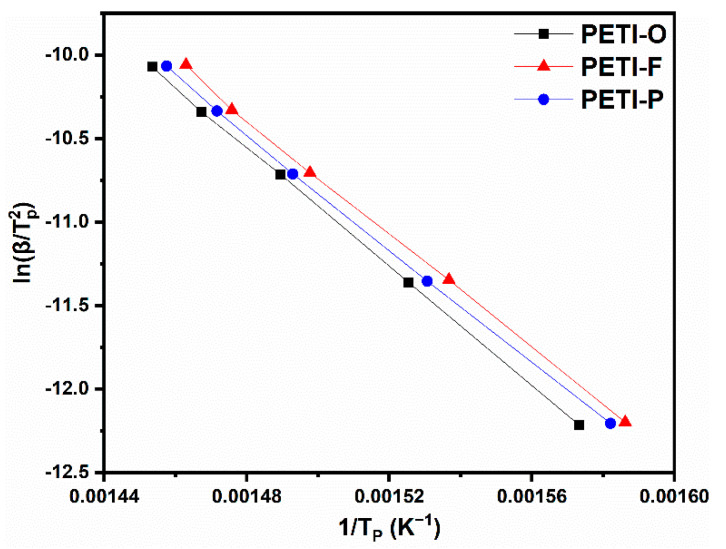
Kissinger plots of PETI-O, PETI-F and PETI-P at T_β._

Nonisothermal DSC method was employed to roughly estimate the activate energy (E_a_) of cure reaction. According to Kissinger’s method, PETI-O, PETI-F and PETI-P were scanned by DSC at different heating rates as described in the experimental section. The Kissinger plot and the calculated E_a_ values are shown in Figure 9 and Table 3. PETI-1 shows the largest E_a_ value of 148.3 kJ/mol, meaning that more active energy was needed for PETI-O when its curing reaction occurs. PETI-O should show the least cure reactivity. The E_a_ values of PETI-F and PETI-P are similar, which showing that they may have similar cure reactivity. The other possible reason is that Kissinger method cannot distinguish the difference of their E_a_ value distinctly because of the error of the Kissinger method [38,54].

**Figure 10:**
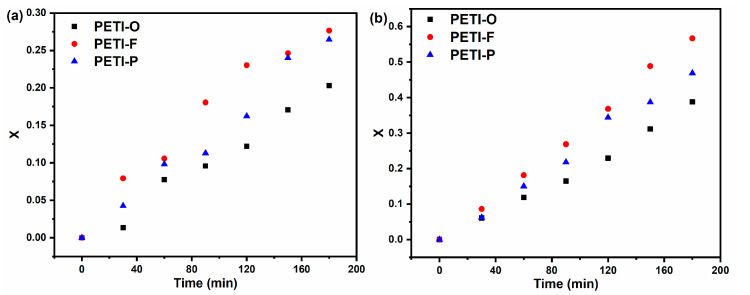
Extent of cure (X) vs. cure time of PETI-O, PETI-F and PETI-P at (**a**) 290 °C and (**b**) 300 °C.

**Figure 11:**
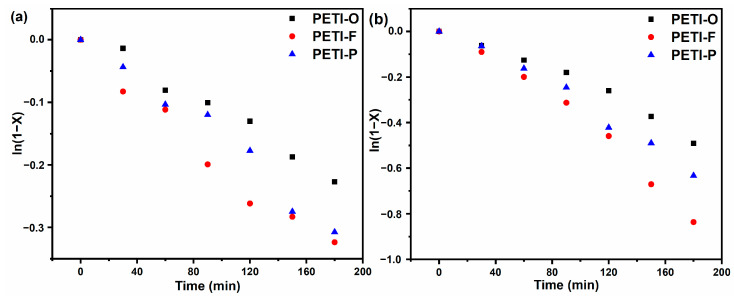
First-order rate plot of the time-dependant disappearance of the phenylethynyl groups at (**a**) 290 °C and (**b**) 300 °C.

The T_g_ values’ change during the isothermal process of 290 or 300 °C for different times were monitored by DSC. The extent of cure (x) of PETI-O, PETI-F and PETI-P were calculated by modified DiBenedetto Equation (2) as described in the experimental section. The T_g_s of materials cured at 380 °C measured by DSC at the heating rate of 40 °C/min, which were defined as T_g__∞_s (Figure S4). Figure 10 shows the cure extent x as a function of isothermal time. With the time of thermal heating at 290 and 300 °C prolonged, extent of cure increases for all the three samples. The curing rate increase in the order of PETI-O < PETI-P < PETI-F. Furthermore, when 0 < x < 0.9, the cure reaction of phenylethynyl groups meets the First-order rate at various temperatures [40]. Figure 11 and Table 3 display the result of the linear fitting, implying that the thermal cure reactions of oligoimides follow the first-order kinetics model. The rate constants of PETI-F with 0.0018 min^−1^ at 290 °C and 0.0047 min^−1^ at 300 °C are larger than them of PETI-O (0.0013 min^−1^ at 290 °C, 0.0026 min^−1^ at 300 °C) and PETI-P (0.0017 min^−1^ at 290 °C, 0.0036 min^−1^ at 300 °C). It is demonstrated again that the cure reaction reactivity of the three samples increases in the order of PETI-O < PETI-P < PETI-F, which is consistent with the result of the above DSC analysis. Because of the faster cure rates of PETI-F and PETI-P, they show worse melting stability than PETI-O. For PETI-1, PETI-2 and PETI-3, it is also found that the more m-PDA units in the polymer backbone, the worse melting stability. However, although PETI-F has higher cure rates than PETI-P, PETI-F has less variation of viscosity than PETI-P. The phenomenon may be related to their difference in melting flow properties and entanglement properties.

#### 3.4.2. Electronic Effect on Reactivity of Phenylethynyl Groups

**Scheme 3:**
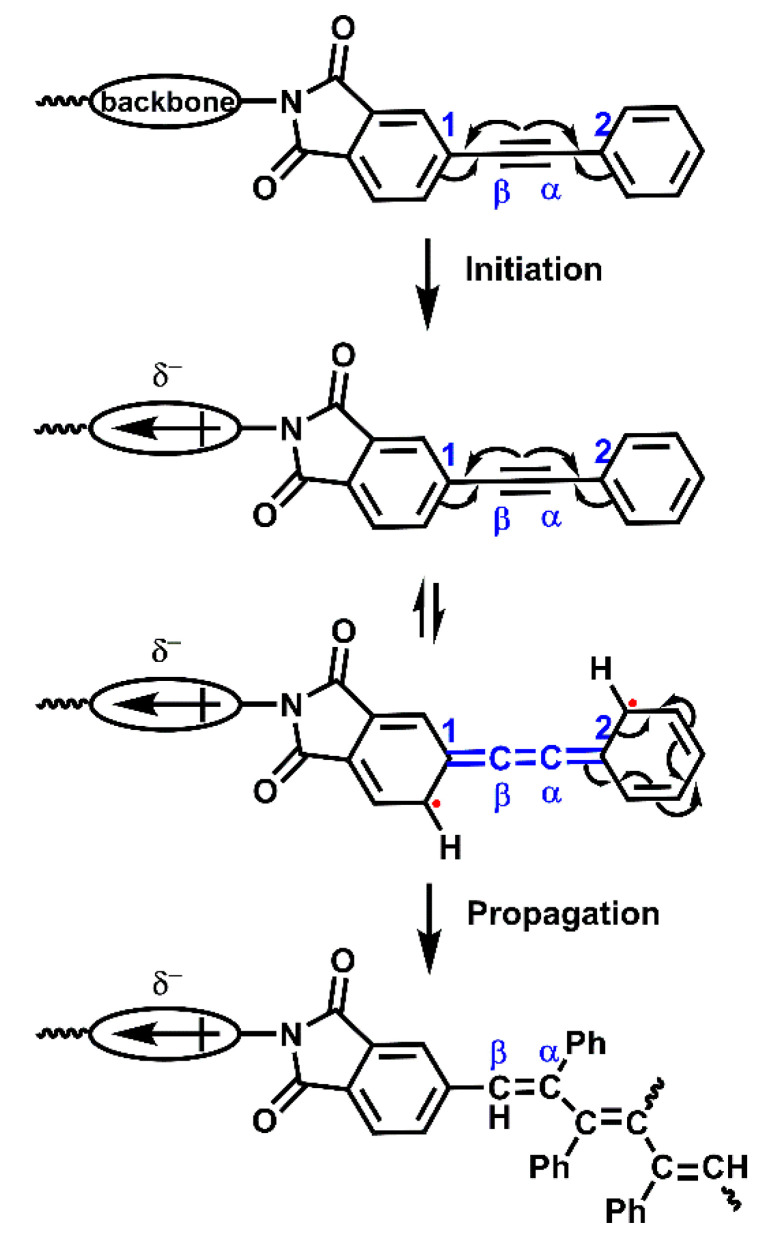
Free radical cure reaction of PEPA-endcapped oligoimides.

According to the previous studies, the thermally induced curing of phenylethynyl groups was thought to be a free radical reaction [55,56], whose process can be simply displayed by Scheme 3.

The initiation of curing reaction occurs via thermally induced generation of free radicals, possibly by cleavage of an ethynyl π-bond, affording an excited state with two unpaired electrons. Then, the propagation proceeds by the addition of the free radical. For the oligoimides in their excited state, unpaired electrons can be stabilized by resonance stabilization of adjacent phenyl rings [57,58]. The resonance stabilization demands overlap of the p orbitals of the one-electron-occupied ethynyl carbons and that of phenyl-carbons that connect to them, which may result in allene-like structure between C_1_ and C_2_ (Scheme 3). As we know, the reactive of free radicals is substantially impacted by the electronic effects of the substituent groups [59,60]_._ For the phenylethynyl-terminated oligoimides in their excited state, electron-donating units in the backbone increase the electron density across the C_β_/C_1_ bond, therefore better facilitate the stabilization of the radical at C_β_ [35]. In this case, the initiation step will be encouraged, whereas the propagation step will be hindered. In contrast to the above situation, electron-withdrawing units/substituents suppress the initiation step and promote the propagation step. According to the previous research [35], when the reaction temperature is high enough so that different PETIs can gain sufficient energy to overcome the activation energy of the initiation step, the rate of cure reaction will be strongly dependent on the propagation step, which will be accelerated by electron-withdrawing units/substituents on the backbone.

As to PETI-O, PETI-F and PETI-P, imide moieties impart electron-withdrawing effect to the phenylethyl groups [20,35]. For PETI-F, the –CF_3_ pendant increases the electron-withdrawing ability of backbone, resulting in the highest cure reactivity of PETI-F. For PETI-O, the -O- linkage of electron-donating ability decrease the electron-withdrawing ability of the backbone, which results in the least cure reactivity of PETI-O. There is no extra electron effect for PETI-P, which impart the medium cure reactivity between PETI-O and PETI-F to PETI-P. This explanation is in good accordance with the study of cure kinetics.

**Figure 12:**
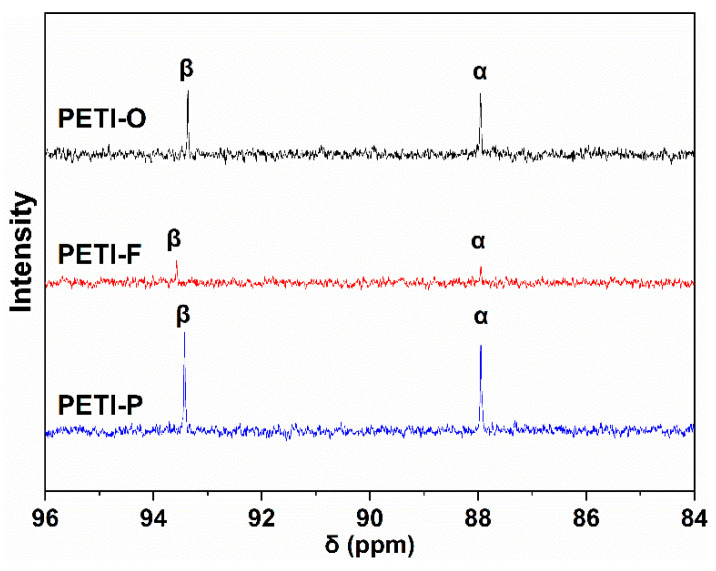
^13^C NMR spectra of PETI-O, PETI-F and PETI-P.

^13^C NMR was used to determine the electron density of C_α_ and C_β_, which indicate the electronic effect of backbone on the terminated phenylethynyl groups. Figure 12 shows the chemical shifts of ethynyl carbon atoms (C_α_ and C_β_) belonging to PETI-O, PETI-F and PETI-P, respectively, and the detailed data are listed in Table 4. As mentioned above, imide units with electron-withdrawing ability shift electron density from terminal phenyl ring (attached to C_α_), onto the C_2_–C_α_ bond while at the same time shift electron density away from the C_1_–C_β_ bond [20,35]. The increased electron-withdrawing ability will intensify the effect, embedding C_β_ with increased deshielding and larger chemical shift while embedding C_α_ with increased shielding and smaller chemical shift. Hence, the difference in chemical shift between the two ethynyl carbons also grows larger. The chemical shifts of C_α_ increase in the order: PETI-F ≈ PETI-P < PETI-O; The chemical shifts of C_β_ increase in the order of PETI-F > PETI-P > PETI-O; The difference of chemical shifts of two ethynyl carbons (C_β_–C_α_) increase in the order of PETI-F > PETI-P > PETI-O. It can be concluded that the electron-withdrawing ability of the backbone of the three PETIs increase in the order: PETI-F > PETI-P > PETI-O, which demonstrate the above explanation.

#### 3.4.3. DFT Study Based on Model Compounds

**Figure 13:**
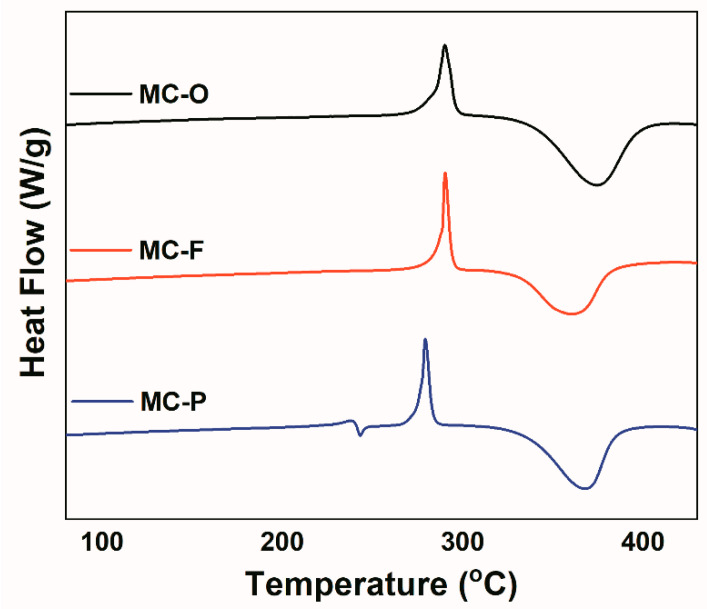
DSC curves of the model compounds.

Density functional theory (DFT) study has widely been used in the research of polyimide [61,62]. Model compounds, MC-O, MC-F and MC-P were prepared in good yield and characterized (Scheme 1). The thermal behavior of the model compounds was investigated by DSC to explore the effect of chemical structures on their thermal curing behavior. Figure 13 shows the DSC curves of the model compounds, and Table 5 lists the DSC data. Both MC-O and MC-F exhibit sharp endothermal peaks at 290 °C, which are assigned as the melt points. However, MC-P shows two endothermal peaks at 238 °C and 279 °C. According to the research of Zhou et al. [47], MC-P experiences a melting-recrystallization-melting process with the ramping of temperature. This multiple melting phenomenon is caused by crystal transition during the heating procedure. The wide exothermic peaks in the range of 332–398 °C are attributed to the thermal crosslinking reactions of phenylethynyl groups. MC-F has curing onset, peak and end temperature of 332, 361 and 382 °C, whose curing reaction occurs at the lowest temperature range. As is expected, the temperature ranges where cure reaction proceeds increase in the order: MC-F < MC-P < MC-O, which is also the order of cure reactivity. The result is in consistence with the analysis of the cure reactivity of PETIs.

**Figure 14:**
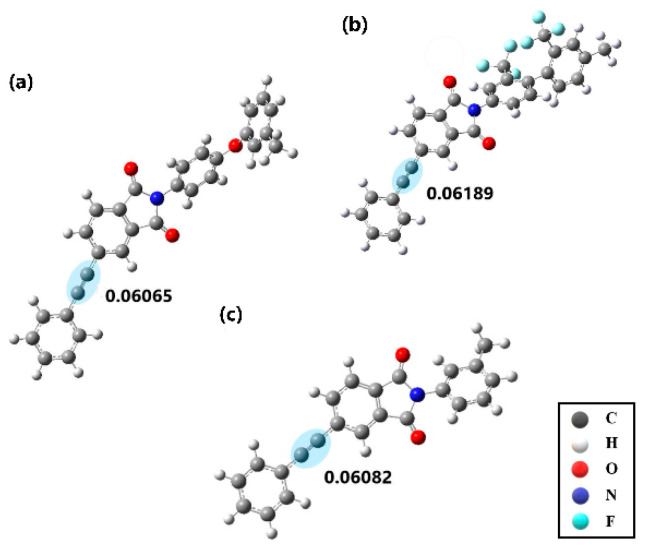
Energy minimized structures and natural bond orbital (NBO) charge of ethynyl group of simplified (**a**) MC–O, (**b**) MC–F and (**c**) MC–P.

In order to further investigate the different reactivity of three model compounds, the electronic structures of model compounds at the ground state and the spin density distribution of the model compounds at the excited state were predicted by the Gaussian09 program. It should be paid attention that the structures of the three model compounds were simplified reasonably for the convenience of calculation. Figure 14 displays the energy minimized structures of the three simplified compounds after geometry optimization. Natural bond orbital (NBO) charge distribution of the model compounds was predicted, which reveals the electron density of each atom or unit [63,64]. The NBO charge values of ethynyl indicate the local electron density of ethynyl units, which are also shown in Figure 14. As is estimated, the values of NBO charge increase in the order: MC-O < MC-P < MC-F, which is caused by the increasing ability of electron-withdrawing of diamine moieties. This result is also consistent with the above ^13^C NMR characterization of PETIs.

**Figure 15:**
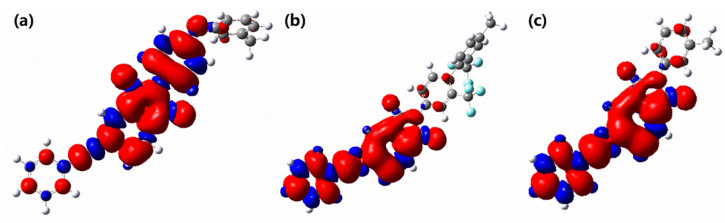
Spin density distribution in (**a**) MC–O, (**b**) MC–F and (**c**) MC–P. The α spin densities are shown in red and the β ones are shown in blue.

Spin density distribution was usually used to study the delocalization of free radicals and to predict their reactivity [65,66,67,68]. For the purpose of characterization of the resonance stabilization of radicals at C_α_ and C_β_, spin density populations of the simplified model compounds at excited state were calculated by Gaussian 09 software (Figure 15); the detailed data of C_α_ and C_β_ are shown in Table 6. The spin density of C_α_ and C _β_ are all far lower than unity, meaning that the radicals are delocalized to the conjugated phenyl rings, imide groups and simplified diamine units. Compared with MC-P and MC-F, ethynyl carbon atoms of MC-O have extremely low spin density with α spin density of 0.067 at C_α_ and β spin density of − 0.039 at C_β_. It is worthy of noting that the oxygen atom of the simplified diamine unit also delocalizes the spin density (Figure 15a). MC-O shows the best resonance stabilization and displays the lowest reactivity. While the radicals of the excited MC-F and MC-P are more local with larger spin density values in C_α_ and C_β_. It is noteworthy that the spin density value of C_α_ of excited MC-F (0.403) is larger than that of excited MC-P (0.397), which reveals the worse resonance stabilization and higher reactivity for the exciting MC-F. It is obvious that the increased electron-withdrawing ability of diamine moieties of the excited model compounds hinders the stabilization of free radicals at C_α_ and C_β_.

In conclusion, the prediction of DFT method indicates that the increased electron-withdrawing ability of oligoimide backbone decreases the electron density of ethynyl units and suppresses the resonance stabilization of free radicals at ethynyl carbon atoms.

### 3.5. Thermal Properties of the Cured Resins

**Figure 16:**
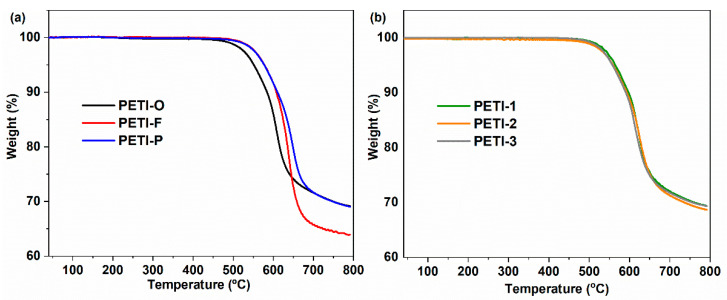
Thermogravimetric analysis (TGA) curves of cured resins in N2 atmosphere; (**a**) curves of cured PETI-O, PETI-F and PETI-P; (**b**) curves of cured PETI-1, PETI-2 and PETI-3.

The synthesized oligoimides were cured at 380 °C for 2 h. The thermogravimetry measurement of the cured resins in N_2_ and under air atmosphere is presented in Figure 16 and Figure S5. The temperature of 5% weight loss (T_d5_) and the char yields at 700 °C are summarized in Table 7. Very small weight loss was detected below 500 °C both in air and N_2_ atmosphere. The thermally cured resins provided with T_d5_ of 558–564 °C under N_2_ and char yields in the range of 63.1–72.0%, which display excellent thermal stability.

**Figure 17:**
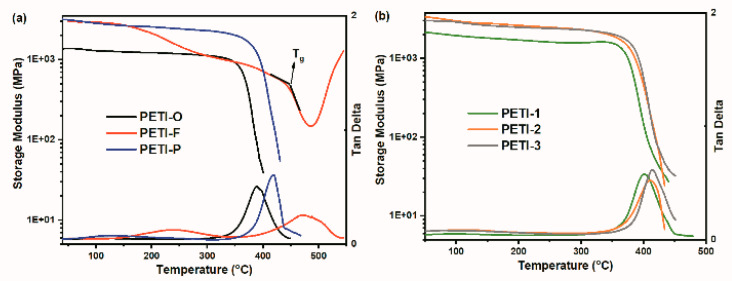
Dynamic mechanical analyses (DMA) curves of cured resins under N2 atmosphere; (**a**) curves of PETI-O, PETI-F and PETI-P; (**b**) curves of PETI-1, PETI-2 and PETI-3.

Figure 17 exhibits the curves of storage modulus and tanδ of the cured resins, and the detailed data are listed in Table 7. In this study, the temperature corresponding to the onset of the decline of storage modulus was defined as T_g_ of cured resins. Because of rigid diamine moieties contained in backbone, cured PETI-F and PETI-P have higher T_g_ values, compared with cured PETI-O (363 °C). It is worth noting that cured PETI-F has the highest T_g_ value of 438 °C, whose DMA curve shows two relaxation processes. TFDB moiety often provides polyimides with high T_g_ value because of their rigid rod-like structure. The two relaxation process is related to the motion of TFDB moieties in the main chains. The low-temperature relaxation process is defined as the sub-glass transition process, and the high-temperature one is defined as glass transition process [43,69].

By copolymerization of 3,4′-ODA and m-PDA, cured PETI-1, PETI-2 and PETI-3 show higher T_g_ values than cured PETI-O. Furthermore, cured resins show higher T_g_ values with the increased content of rigid m-PDA moiety. For instance, cured PETI-3 synthesized by 3,4′-ODA and m-PDA content at a mole ratio of 50:50 has the T_g_ value of 391 °C, 9 °C higher than that of cured PETI-2 (382 °C), and 11 °C higher than that of cured PETI-1 (380 °C). While synthesized by homopolymerization of m-PDA, cured PETI-P has T_g_ value of 398 °C, 7 °C higher than that of cured PETI-3.

### 3.6. Mechanical Properties of the Thermal-Cured Resins

Table 8 summarizes the mechanical properties of the cured resins. After thermal curing at 380 °C for 2 h, all cured resins exhibit good mechanical properties with the tensile strength of 54.2–66.2 MPa, tensile modulus of 1.96–2.40 GPa, and elongation at breakage of 2.5–3.4%. The flexural strength and modulus of the resins are 126–137 M Pa and 3.1–3.6 GPa, respectively. Compared with previous research [37,47], all the cured resins show low strength and toughness because of their high crosslink density. The thermal-cured PETI-1, PETI-2 and PETI-3 do not show apparent difference in mechanical compared with PETI-O and PETI-P, revealing that copolymerization did not exert obvious impacts on mechanical properties of thermally cured resins.

## 4. Conclusions

A series of phenylethynyl-endcapped polyimides with a calculated molecular weight of 1000 g/mol based on 6FDA was prepared, whose thermal property, rheology behavior and mechanical property were characterized. This study has found that PETIs copolymerization by 3,4′-ODA and m-PDA gain excellent balance between processability, mechanical property and heat-resistance properties. The rigid m-PDA units endow oligoimides’ backbone with meta catenation, resulting in lower crystalline, lower melting temperature, wider processing temperature window and higher cured T_g_ values. Meanwhile, copolymerization does not exert negative effect on mechanical property of the cured resins. PETI-O and PETI-1 are all amenable to RTM. In particular, PETI-1 has low processing temperature (280 °C) and high T_g_ of 380 °C. Its neat-cured resin has good mechanical properties with tensile strength of 62.2 MPa and elongation at breakage of 3.4%.

The curing reaction of phenylethynyl groups proceeds during isothermal heating, which leads to the variation of melt viscosity. The melting stabilities of oligoimides are substantially related to their curing rates, melting flow properties and the entanglement properties. Herein, the electronic effect of backbone on the curing rates was investigated. By the method of DSC, ^13^C NMR and DFT, it was confirmed that the increased electron-withdrawing ability of oligoimides backbone results in higher reactivity of terminated phenylethynyl groups and worse melting stability. The trifluoromethyl (−CF_3_) groups attached to backbones increase the electron-withdrawing ability of oligoimide backbone; Ether linage (–O–) on the backbones, decrease the electron-withdrawing ability of oligoimide backbone. The curing reactivity of terminated phenylethynyl groups increases in the order of PETI-O < PETI-P < PETI-F. PETI-F and PETI-P also gain worse melting stability. The electron-withdrawing ability of oligoimide backbones plays an important role in the melting stability of phenylethynyl end-capped oligoimides.

## Figures and Tables

**Table 1:** Physical properties of uncured oligoimides.

Sample	GPC			DSC ^a^				
	M_n_	M_w_	M_w_/M_n_	T_g_ (°C)	T_m_ (°C)	ΔH_endo_ (J/g)	T_onset_ (°C)	T_exo_ (°C)
PETI-O	1346	1986	1.48	157	215, 246	38.7	361	402
PETI-F	1303	1831	1.41	170	214, 272	25.9	350	391
PETI-P	1333	1915	1.44	158	-	-	360	395
PETI-1	1333	1940	1.46	147	216, 245	20.5	357	398
PETI-2	1287	1861	1.45	148	215, 242	18.6	361	401
PETI-3	1302	1823	1.40	144	242	6.30	359	397

^a^ Measurement were performed by first differential scanning calorimetry (DSC) scan at a rate of 10 °C/min; T_g_: glass transition temperature; T_m_: peak temperature of melting endothermal peaks; ΔH_endo_ (J/g): melting enthalpy; Tonset: onset temperature of exothermal behavior; Texo: Temperature of exothermic peaks on DSC curves.

**Table 2:** Complex viscosity data of PETIs.

Sample	ΔT_|η*|≤1Pa∙s_^a^ (°C)	|η*|min ^b^ (Pa·s)	Melt Viscosity Variation for 2 h ^c^		
			Δ|η*|_280 °C_ (Pa·s)	Δ|η*|_290 °C_ (Pa·s)	Δ|η*|_300 °C_ (Pa·s)
PETI-O	95	0.15 (at 309 °C)	0.52–2.17	0.20–0.70	0.17–1.13
PETI-F	65	0.31 (at 331 °C)	2.41–38.9	0.69–32.0	0.50–3492
PETI-P	65	0.45 (at 323 °C)	1.52–19.1	0.88–930	0.66–8548
PETI-1	102	0.16 (at 312 °C)	0.31–0.94	0.22–2.83	0.14–588
PETI-2	104	0.18 (at 312 °C)	0.35–1.31	0.25–3.56	0.19–1070
PETI-3	105	0.16 (at 315 °C)	0.46–1.72	0.30–9.88	0.23–1185

^a^ T_|η*|≤1 Pa__∙__s_: temperature scale regard to |η*| ≤ 1 Pa∙s. ^b^ |η*|_min_: minimum melt viscosity and temperature at this value. ^c^ Melt viscosity variation for 2 h: melt viscosity variation of samples during isothermal rheological measurement at different temperature for 2 h; For example, Δ|η*|_280 °C_ means experimental data recorded at 280 °C.

**Table 3:** Kinetic parameters of PETI-O, PETI-F and PETI-P.

Sample	E_a_(kJ/mol)	290 °C		300 °C	
		Rate Constant (min^−1^)	Regression Coefficient	Rate Constant (min^−1^)	Regression Coefficient
PETI-O	148.3	0.0013	0.978	0.0026	0.970
PETI-F	142.7	0.0018	0.970	0.0047	0.975
PETI-P	142.2	0.0017	0.970	0.0036	0.983

**Table 4:** Chemical shifts of ethynyl carbons of PETI-O, PETI-F and PETI-P.

	C_α_	C_β_	C_β_–C_α_
PETI-O	87.96	93.37	5.41
PETI-F	87.95	93.57	5.62
PETI-P	87.95	93.43	5.48

**Table 5:** Thermal data of the model compounds.

Sample	Endothermal Temperature (°C)	Exothermal Temperature (°C)		
	Peak	Onset	Peak	End
MC-O	290	339	374	398
MC-F	290	332	361	382
MC-P	238, 279	335	368	383

**Table 6:** The spin density distribution of the three simplified model compounds.

	MC-O	MC-F	MC-P
C_α_	0.067	0.403	0.397
C_β_	−0.039	−0.002	0.003

**Table 7:** Thermal properties of the thermal-cured polyimides cured at 380 °C for 2 h.

Sample	T_g_^a^ (°C)	N_2_^b^		Air ^c^	
		T_d5_^b^ (°C)	Char Yield ^c^ (%)		T_d5_ (°C)
PETI-O	363	558	71.6		558
PETI-F	438	578	65.8		578
PETI-P	398	577	71.7		578
PETI-1	380	564	72.0		565
PETI-2	382	559	71.2		557
PETI-3	391	558	71.7		558

^a^ T_g_: Determined by DMA at a heat rate of 5 °C/min at 1 Hz under N_2_ atmosphere. ^b^ Measurement were performed at a heat rate of 20 °C/min under N_2_ atmosphere; T_d5_ means temperature at weight loss of 5%; Char yield: percent of weight maintenance at 700 °C. ^c^ Measurement were performed at a heat rate of 20 °C/min under air atmosphere.

**Table 8:** Mechanical properties of the cured resins.

Sample	Tensile Properties			Flexural Properties	
	Strength (MPa)	Modulus (GPa)	Elongation (%)	Strength (MPa)	Modulus (GPa)
PETI-O	54.2 (±4.86)	1.99 (±0.08)	2.8 (±0.54)	125.6 (±1.57)	3.1 (±0.07)
PETI-F	66.2 (±5.67)	2.40 (±0.10)	3.1 (±0.60)	126.2 (±5.39)	3.6 (±0.08)
PETI-P	64.0 (±5.23)	2.28 (±0.11)	2.9 (±0.69)	132.3 (±8.90)	3.2 (±0.18)
PETI-1	62.2 (±5.12)	1.96 (±0.07)	3.4 (±0.32)	136.2 (±6.34)	3.2 (±0.14)
PETI-2	50.8 (±6.75)	2.07 (±0.03)	2.5 (±0.37)	128.8 (±7.25)	3.3 (±0.09)
PETI-3	56.4 (±3.17)	2.07 (±0.07)	2.9 (±0.25)	137.1 (±7.01)	3.2 (±0.04)

## Data Availability

No new data were created or analyzed in this study. Data sharing is not applicable to this article.

## Supplementary Materials

The following are available online at https://www.mdpi.com/2073-4360/13/6/903/s1, Figure S1: (a) ^1^H and (b) ^13^C NMR spectra of PETI-O in DMSO-d6, Figure S2: (a) ^1^H and ^13^C NMR spectra of PETI-F in DMSO-d_6_, Figure S3: (a) ^1^H and (b) ^13^C NMR spectra of PETI-P in DMSO-d_6_, Figure S4: DSC plot of PETI-O, PETI-F and PETI-P cured at 380 °C/2 h, Figure S5: TGA curves of cured resins in air atmosphere, Table S1: Molecular structures of the chemical species detected by MALDI-TOF for oligoimides.
