# Supplementary material for: Resin Transfer Moldable Fluorinated Phenylethynyl-Terminated Imide Oligomers with High Tg: Structure–Melt Stability Relationship
Source: Polymers (Basel). 2021 Mar 15;13(6):903. doi: 10.3390/polym13060903 (PMC7999610; doi:10.3390/polym13060903)
Supplement: Supplementary file 1 [file polymers-13-00903-s001.pdf]

polymers

MDPI

Supporting information

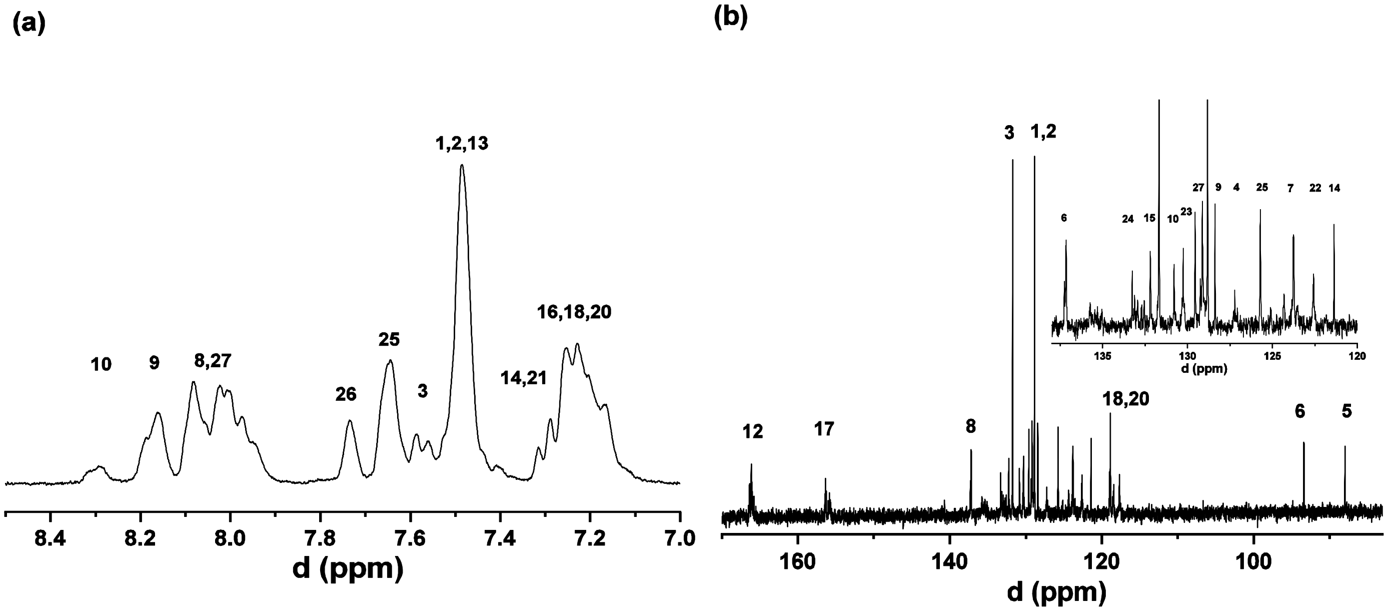

Figure 1. (**a**) ^{1}H and (**b**) ^{13}C NMR spectra of PETI-O in DMSO-d_6.

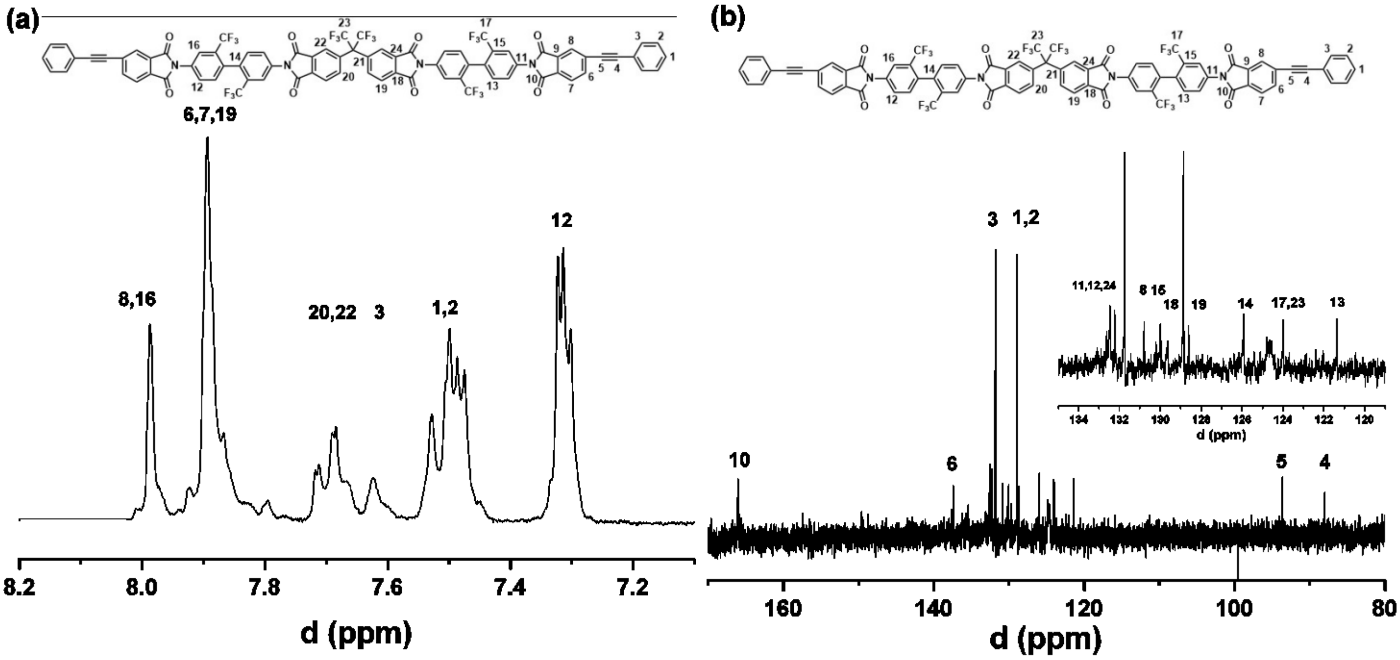

Figure S2. (**a**) ^{1}H and (**b**) ^{13}C NMR spectra of PETI-F in DMSO-d_6

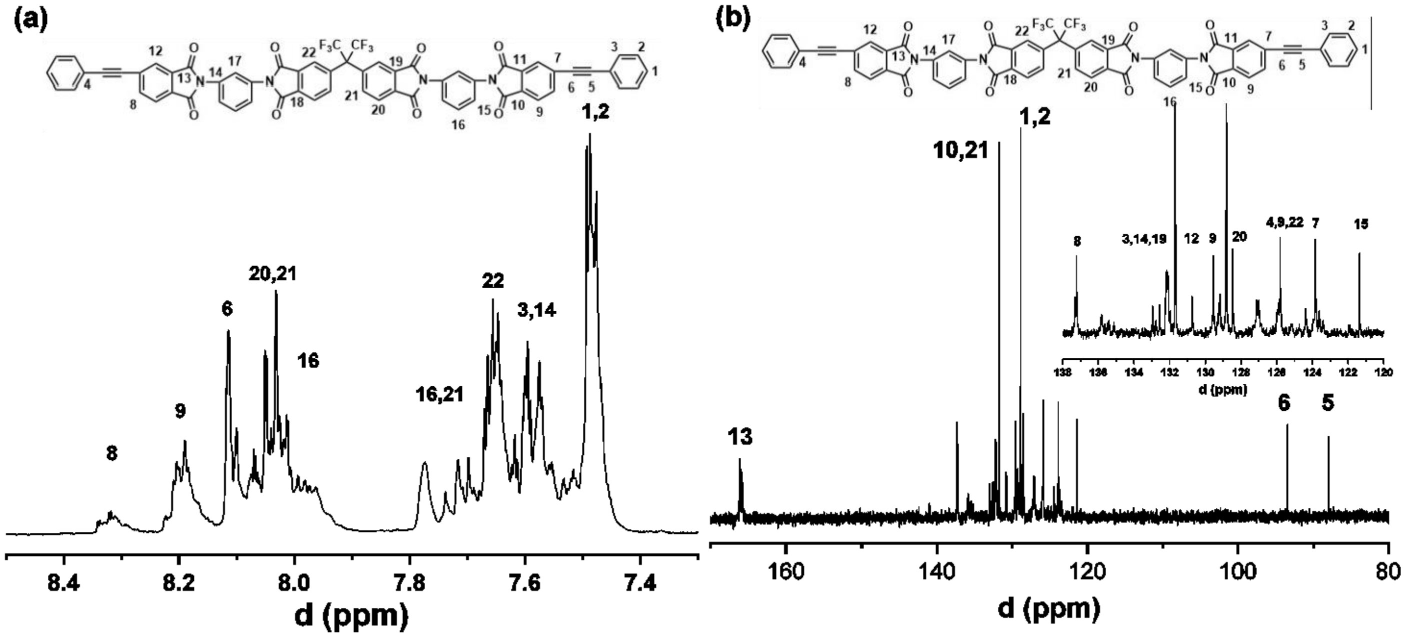

Figure S3. (**a**) ^{1}H and (**b**) ^{13}C NMR spectra of PETI-P in DMSO-d_6

Table 1. Molecular structures of the chemical species detected by MALDI-TOF for oligoimides.

	Chemical structures of the oligoimide species	n	Mass(m/z)
**PETI-O**	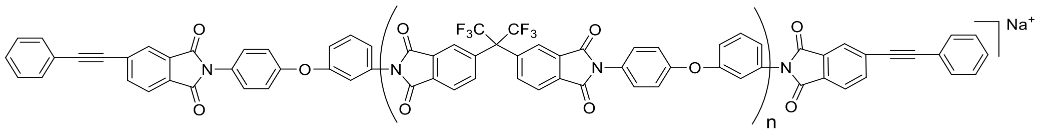	0	683
		1	1291
		2	1899
		3	2507
**PETI-F**	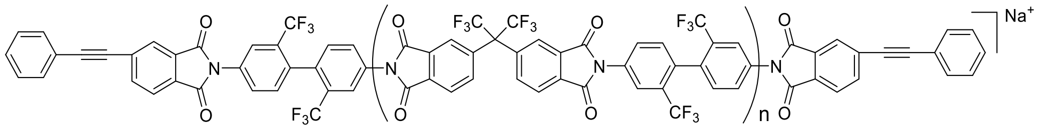	0	804
		1	1532
		2	2261
		3	2989
**PETI-P**	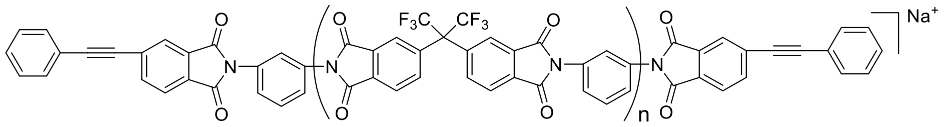	0	591
		1	1108
		2	1624
		3	2141

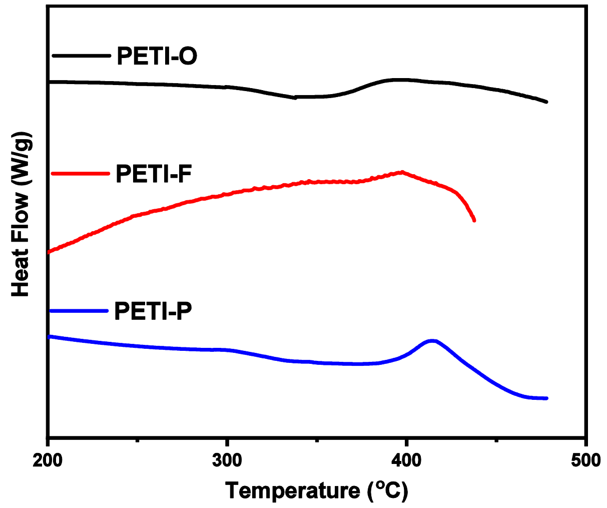

Figure S4. DSC plot of PETI-O, PETI-F and PETI-P cured at 380 °C/2h

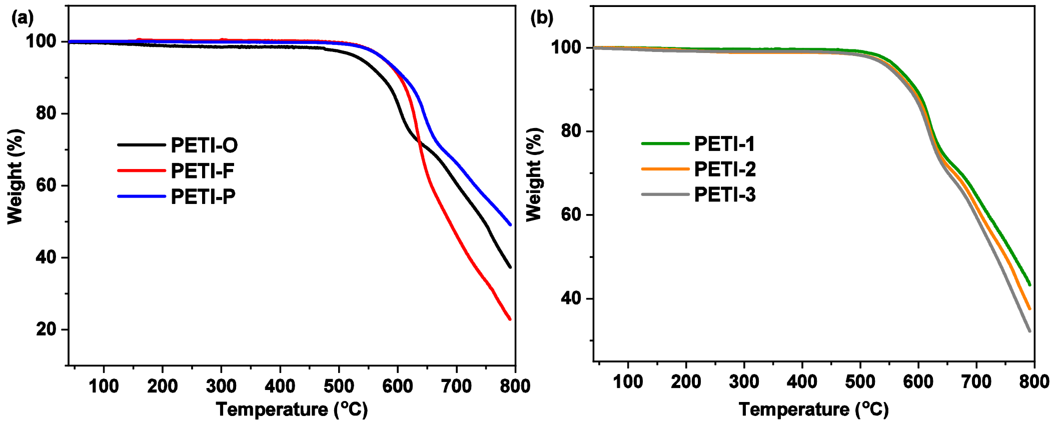

Figure S5. TGA curves of cured resins in air atmosphere
